# H-Shaped Radial Phononic Crystal for High-Quality Factor on Lamb Wave Resonators

**DOI:** 10.3390/s23042357

**Published:** 2023-02-20

**Authors:** Weitao He, Lixia Li, Zhixue Tong, Haixia Liu, Qian Yang, Tianhang Gao

**Affiliations:** 1 School of Mechanical and Electrical Engineering, Xi’an University of Architecture and Technology, Xi’an 710055, China; 2 Institute of Mechanics, Xi’an University of Architecture and Technology, Xi’an 710055, China

**Keywords:** radial phononic crystal, complex band curve, MEMS resonator, quality factor, anchor loss

## Abstract

In this paper, a novel H-shaped radial phononic crystal (H-RPC) structure is proposed to suppress the anchor loss of a Lamb wave resonator (LWR), which has an ultra-high frequency (UHF) and ultra-wideband gap characteristics. Compared to previous studies on phononic crystal (PC) structures aimed at suppressing anchor loss, the radial phononic crystal (RPC) structure is more suitable for suppressing the anchor loss of the LWR. By using the finite element method, through the research and analysis of the complex energy band and frequency response, it is found that the elastic wave can generate an ultra-wideband gap with a relative bandwidth of up to 80.2% in the UHF range when propagating in the H-RPC structure. Furthermore, the influence of geometric parameters on the ultra-wideband gap is analyzed. Then, the H-RPC structure is introduced into the LWR. Through the analysis of the resonant frequency, it is found that the LWR formed by the H-RPC structure can effectively reduce the vibration energy radiated by the anchor point. The anchor quality factor was increased by 505,560.4% compared with the conventional LWR. In addition, the analysis of the LWR under load shows that the LWR with the H-RPC structure can increase the load quality factor by 249.9% and reduce the insertion loss by 93.1%, while the electromechanical coupling coefficient is less affected.

## 1. Introduction

In recent years, with the rapid development of the fifth-generation mobile communication network (5G), microelectromechanical system (MEMS) technology has shown good application prospects in wireless communication systems and sensor networks [1,2]. The Lamb wave resonator (LWR) is considered by most researchers to be the preferred choice for miniaturized, high-performance, and low-power integrated resonators due to its high operating frequency, high electromechanical coupling coefficient, and low power consumption [3,4]. LWRs are considered to be highly desirable in the 300–800 MHz range commonly used in the field of wireless communications [2,5,6]. Studies have shown that LWRs with a high quality factor (Q) can achieve lower insertion loss filters and high-resolution sensors [7,8].

It has been reported that the Q of LWRs can be effectively improved by reducing anchor loss [9,10,11,12,13,14,15]. Acoustic energy radiates to the fixed area through the support tether [16]. Harrington et al. improved the quality factor to 12,042 by applying acoustic reflectors on the substrate [9]. Zou et al. demonstrated a butterfly resonator to reduce the vibration around the anchor [17]. Pandey et al. designed a mesa around the resonator to reflect the elastic energy back to the resonator [18]. Using the band gap characteristics of phononic crystals (PCs) to suppress the anchor loss has attracted the attention of researchers [3,11,15,19,20,21,22,23,24,25,26]. For example, Zhu et al. used a two-dimensional stomatal PCs unit cell to increase the Q to twice the original [11]. Ardito et al. used a one-dimensional PCs structure to increase the Q by several times [24]. Other shapes, such as rings [27], cross-like holes [13], fractals [14], Spider Web-Likes [15], cross-section connections [25], double “I” holes [26], and snowflakes [28] have also been reported. At present, Yinjie Tong has increased the Q of Pillar-Based PCs by 54% in the UHF range [3]. Although the Pillar-Based PCs improve the Q, they have a narrow band gap, a relatively low bandwidth, and a limited application frequency band.

The radial phononic crystal (RPC) structure is an annular structure arranged periodically along the cylindrical coordinate system with complete band gap characteristics [29,30,31,32,33]. Torrent et al. proposed a new shape of a RPC structure for the first time and verified the existence of its acoustic band gap [29]. A new shape of a double-layer RPC was designed by Ma et al., and the cause of the band gap was explained by its propagation characteristics [32]. Shi et al. designed different two-dimensional radial periodic structures to achieve low-frequency band gaps [34]. The usual design for one-dimensional PCs is to arrange them on the LWR support tether or in a two-dimensional arrangement on the anchor [15,16,19,22,25,26,27,35,36,37,38,39]. In the case of one-dimensional PCs, they are often arranged periodically along the *x*-axis on the support tether [16,22,35,36,37,38,39]. Therefore, one-dimensional PCs can effectively block the propagation of sound waves only by satisfying the directional band gap on the Г-X, but they also require a longer support tether to arrange PCs. Two-dimensional PCs are often arranged periodically on the anchor along the *x*-axis and *y*-axis [15,19,22,25,26,27,35,38]. Due to the arrangement of two-dimensional PCs, to effectively block the propagation of acoustic waves it is necessary to have directional band gaps along the Г-X, X-M, and M-Г directions of the irreducible Brillouin zone to achieve omnidirectional band gaps. When an RPC with a Γ-R band gap is applied to the resonator anchor it can completely block the propagation of sound waves.

In this paper, an H-shaped radial phononic crystal (H-RPC) structure applied to an LWR is proposed. The H-RPC structure is composed of an H-shaped plane rotation. In the second part, we show the H-RPC structure model and how to calculate the complex band curve of the RPC structure by a theoretical method. In the third part, the complex band curve and frequency response curve of the structure is calculated by using the finite element method (FEM). The influence of different periods on the attenuation effect is studied in depth, and the influence of different geometric parameters on the ultra-wideband band gap is analyzed. In the fourth part, an H-RPC is applied to an LWR, and the influence of different spacing between the H-RPC and the support tether on the Q, electromechanical coupling coefficient, and insertion loss is analyzed. Finally, a brief conclusion is arranged. The main contribution of this paper is to introduce the RPC structure into the LWR, which produces an ultra-wideband gap and is suitable for a short support tether LWR.

## 2. Materials and Methods

### 2.1. Radial Phonon Crystal Model

**Figure 1:**
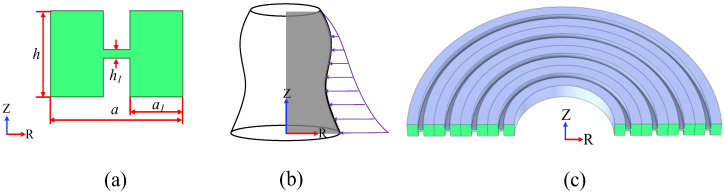
(**a**) The cross section of H-RPC unit cell; (**b**) The formation of RPC; (**c**) The three-dimensional model formed by the rotation of 4-cycle H-RPC structure 180°.

In this study, the proposed H-RPC structure is composed of centrosymmetric grooves, as shown in Figure 1. Figure 1a shows the H-RPC unit cell section diagram, Figure 1b shows the unit cell rotating around the *z*-axis diagram, and Figure 1c shows the three-dimensional H-RPC structure. The lattice constant a=8 μm, the total height h=5 μm, the left and right-side wall width $a{\;}_{1}=3.2\;\mu m$, and the middle support beam height $h_{1}=0.8\;\mu m$. Since the structure is obtained by rotating around the Z-direction, the irreducible Brillouin zone of the three-dimensional H-RPC is the Г-R region. The H-RPC is composed of commonly used anisotropic monocrystalline silicon. Among them, the density (ρ) is 2330 kg/m^3^, and the elastic modulus E of anisotropic single-crystal silicon, shear modulus G, and Poisson’s ratio σ are shown in Table 1.

### 2.2. Theoretical Method

The H-RPC studied adopts a two-dimensional axisymmetric finite element method based on a cylindrical coordinate system. Therefore, the traditional Cartesian coordinate system is transformed into an elastic wave Equation (1) in a cylindrical coordinate system, and the energy band curve of the RPC in an infinite period is further studied.

(1) $$\begin{array}{c} \{\begin{array}{l} \rho \frac{\partial^{2}u}{\partial t^{2}}=\left(\lambda +2\mu \right)\frac{\partial \theta_{t}}{\partial r}-\frac{2\mu }{r}\frac{\partial w_{z}^{\text{’}}}{\partial \theta }+2\mu \frac{\partial w_{\theta }^{\text{’}}}{\partial z}\\ \rho \frac{\partial^{2}v}{\partial t^{2}}=\left(\lambda +2\mu \right)\frac{\partial \theta_{t}}{r\partial \theta }-2\mu \frac{\partial w_{z}^{\text{’}}}{\partial z}+2\mu \frac{\partial w_{r}^{\text{’}}}{\partial r}\\ \rho \frac{\partial^{2}w}{\partial t^{2}}=\left(\lambda +2\mu \right)\frac{\partial \theta_{t}}{\partial z}-\frac{2\mu }{r}\frac{\partial }{\partial r}\left(rw_{\theta }^{\text{’}}\right)+\frac{2\mu }{r}\frac{\partial w_{r}^{\text{’}}}{\partial \theta } \end{array} \end{array}$$

In Equation (1), *u, v,* and *w* are the displacement components of the Cartesian coordinate system, *ρ* is the density, *t* is time, the elastic wave constants of *λ* and *μ* are the materials, and *r, θ,* and *z* are the coordinate displacement components of the cylindrical coordinate system. The volumetric strain *θ_t_* and the rotating component ($w_{r}^{\text{′}}$,$w_{\theta }^{\text{′}}$, $w_{z}^{\text{′}}$) are defined as:

(2) $$\begin{array}{c} \{\begin{array}{l} \theta_{t}=\frac{1}{r}\frac{\partial \left(ru\right)}{\partial r}+\frac{1}{r}\frac{\partial v}{\partial \theta }\frac{\partial w}{\partial z}\\ w_{r}^{\text{’}}=\frac{1}{2}\left(\frac{1}{r}\frac{\partial w}{\partial \theta }+\frac{1}{r}\frac{\partial v}{\partial z}\right)\\ w_{\theta }^{\text{’}}=\frac{1}{2}\left(\frac{1}{r}\frac{\partial u}{\partial \theta }+\frac{\partial w}{\partial r}\right)\\ w_{z}^{\text{’}}=\frac{1}{2}\left(\frac{1}{r}\frac{\partial \left(rv\right)}{\partial \theta }+\frac{\partial u}{\partial \theta }\right) \end{array} \end{array}$$

because the RPC structure is arranged so that the lattice unit is infinite along the radial direction. According to the Bloch theorem, only one lattice unit is needed. The lattice boundary condition equation is:

(3) $$\begin{array}{c} u\left(r+r_{a},z\right)=u\left(r,z\right)e^{ik_{r}r_{a}} \end{array}$$

where *r* is the radial position, *a* is the lattice constant, and $k_{r}$ is the radial component of the Bloch wave vector Kr.

Figure 1a is established under the 2D axisymmetric component of COMSOL Multiphysics, and the periodic boundary conditions of Formula (3) are applied in the R-direction. By scanning the Bloch wave vector Kr (the real part and the imaginary part of the wave vector) of the first irreducible Brillouin zone boundary in the R-direction as shown in Figure 1a, the complex band curve of the RPC structure can be obtained.

## 3. Ultra-Wideband Gap Characteristics of H-RPC

### 3.1. Ultra-Wideband Gap Structure

**Figure 2:**
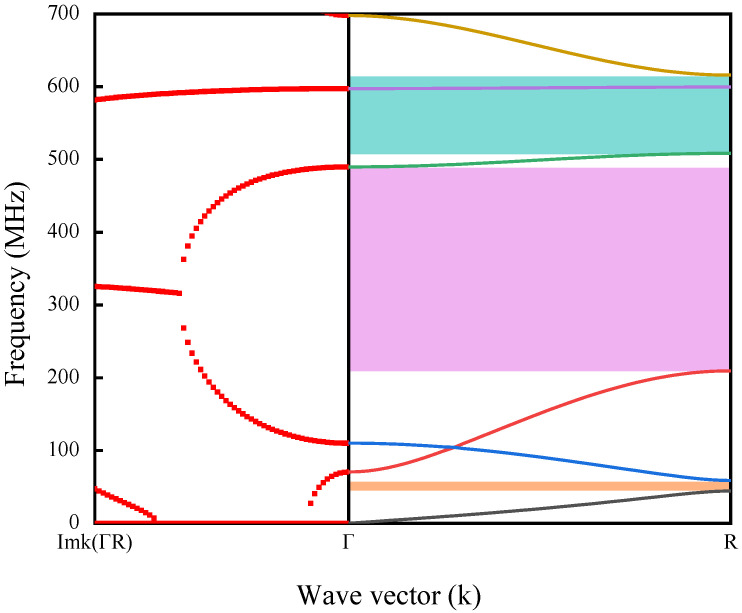
Complex energy band curve of the H-RPC structure. The real wave vector energy band curve in (Г-R) direction is represented by solid lines of different colors on the right side, and the imaginary wave vector energy band curve in (Г-R) direction is represented by red dotted lines on the left side.

The complex band curve of the H-RPC structure is shown in Figure 2. In Figure 2, the real wave vector energy band curve in (Г-R) direction is represented by solid lines of different colors on the right side, and the imaginary wave vector energy band curve in (Г-R) direction is represented by red dotted lines on the left side. If there is a frequency range where no band curve is present in the real wave vector energy band curve, then this is a complete band gap. When the frequency is in the band gap range, the absolute value of the imaginary wave vector can be used to represent the attenuation characteristics in the band gap. The larger the absolute value of the imaginary wave vector, the stronger the attenuation. It can be seen from the figure that six real wave energy vector band curves are found in 0–700 MHz, and three complete band gaps are generated (as shown in the shadow on the right side of Figure 2). The first complete band gap is obtained at 44.3–58.7 MHz, which is generated between the first energy band curve and the second energy band curve. A second complete band gap is obtained at 209.4–489.7 MHz, which is generated between the third energy band curve and the fourth energy band curve. The third complete band gap is obtained at 508.3–615.8 MHz, which is generated between the fourth energy band curve and the sixth energy band curve. From the energy band curve of the imaginary wave vector, it can be observed that when the frequency is in the second band gap, the absolute value of the imaginary wave vector shows a continuous and stable change, which is mainly caused by the Bragg mechanism.

Using the relative bandwidth BG to measure the utilization and availability of RPCs, the relative bandwidth can be calculated by the following formula:

(4) $$\begin{array}{c} BG\%=\frac{\left(f_{2}-f_{1}\right)}{\left(\frac{f_{2}+f_{1}}{2}\right)}\% \end{array}$$

where $f_{1}$ is the starting frequency of the band gap and $f_{2}$ is the cutoff frequency of the band gap. From the complex band curve of Figure 2, it can be seen that the first band gap corresponds to $BG_{1}=28.0\%$, the second band gap corresponds to $BG_{2}=80.2\%$, and the third band gap corresponds to $BG_{3}=19.1\%$. In particular, the ultra-wideband gap in the UHF range is achieved in $BG_{2}$. Compared with the 24.1% relative bandwidth of the cylindrical PCs proposed in the UHF range, the relative bandwidth of the band gap generated by the H-RPC structure proposed in this paper is as high as 80.2% [3]. Table 2 compares the band gaps of similar lattice constant structures in the relevant literature.

### 3.2. Frequency Response

**Figure 3:**
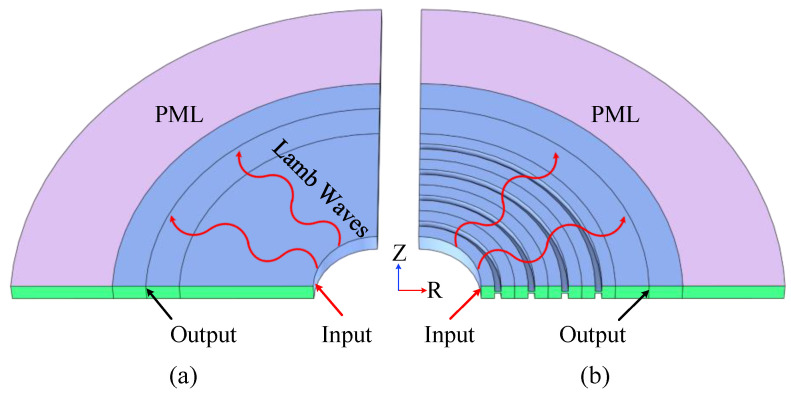
Frequency response model. (**a**) is a traditional model; (**b**) is a 4-period H-RPC model.

In order to verify the stopband effect of the radial structure a comparative analysis of different periodic models is established, and the frequency response curve (the transfer function is defined to be equal to $20log\;\left(\alpha_{output}/\alpha_{input}\right)$, and $\alpha_{output}$ and $\alpha_{input}$ are respectively expressed as the acceleration of output and input) is used to measure the degree of the stopband. Specifically, the frequency response model is established as shown in Figure 3, while Figure 3a shows the traditional contrast propagation model and Figure 3b shows the H-RPC model with four cycles. As shown in Figure 3, an R-direction displacement excitation is applied to the input probe and the output probe is used to pick up the displacement results. In order to reduce the reflection of elastic waves and interfere with the propagation process, a perfect matching layer (PML) is set at both ends of the model.

**Figure 4:**
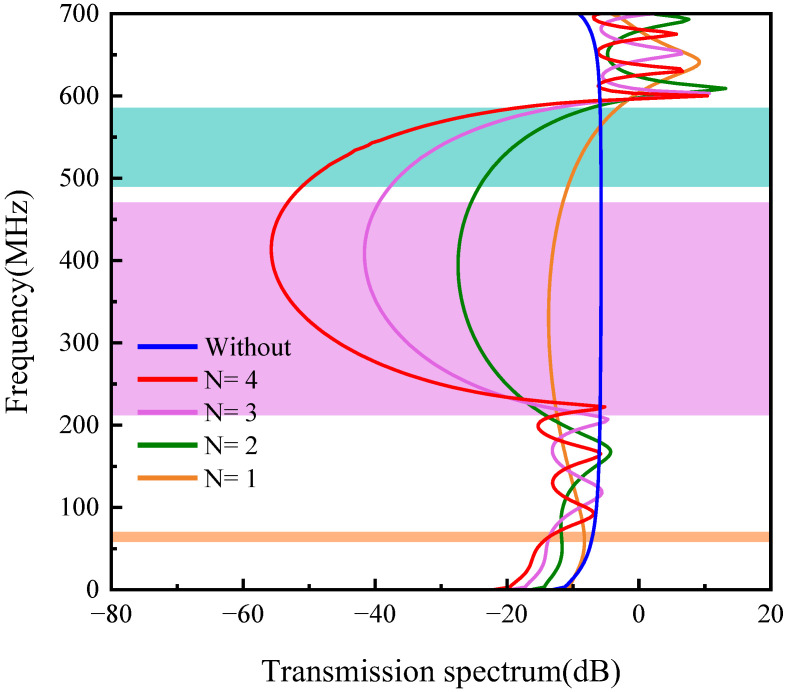
H-RPC frequency response curve.

The frequency response curve is shown in Figure 4. In the first band gap (44.3–58.7 MHz), the frequency transmission response of the PnC model is lower than that of the comparison model. In the second band gap (209.4–489.7 MHz) and the third band gap (508.3–615.8 MHz), the model composed of PCs is significantly lower than the corresponding model. When the frequency is about 400 MHz, the maximum attenuation is 55 dB. When the frequency is in the first band gap range, the attenuation does not decrease significantly. This is because the wavelength of the elastic wave is much larger than the unit cell of the PnC when in the first band gap range. At this time, although the band gap appears in the band structure, it does not play a strong role in the simulation application. It is particularly noteworthy that with the increase of the period N, the attenuation in the second band gap and the third band gap will gradually increase. This is because with the increase of the period the elastic wave will be attenuated when passing through the PnC of each period.

### 3.3. The Influence of Geometric Parameters on Ultra-Wideband Band Gap

**Figure 5:**
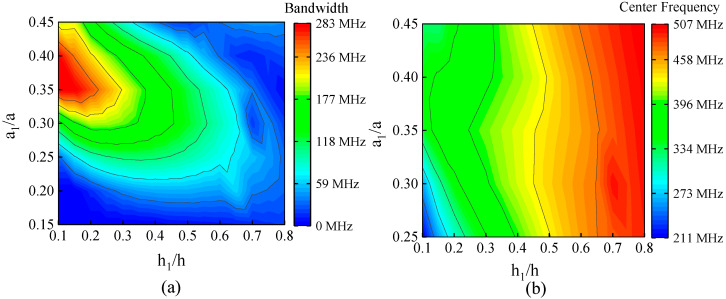
The influence of geometric parameters on ultra-wideband gap. (**a**) the influence of geometric parameter ratio ($h_{1}/h$, $a_{1}/a$) on bandwidth; (**b**) the influence of geometric parameter ratio ($h_{1}/h$, $a_{1}/a$) on center frequency.

For the formation of an ultra-wide band gap, the geometric parameters of the unit cell play a fundamental role. Therefore, in this section, the influence of the geometric parameters $a_{1}$ and $h_{1}$ of the structure on the ultra-wideband gap is discussed. Under the condition of keeping the lattice constant a and height h unchanged, the influence of changing the value of $h_{1}/h$ and $a_{1}/a$ on the ultra-wideband gap is shown in Figure 5. In Figure 5, Figure 5a shows the variation of the bandwidth of the ultra-wideband gap with $h_{1}/h$ and $a_{1}/a$, and Figure 5b shows the variation of the center frequency of the ultra-wideband gap with $h_{1}/h$ and $a_{1}/a$. As $h_{1}/h$ increases, the band gap width gradually decreases to zero, and the center frequency of the band gap gradually shifts to high frequency. This is because as $h_{1}$ gradually increases, the propagation obstacle of the elastic wave at the connection boundary of the support beam decreases. With the increase of $a_{1}/a$, the band gap width increases first and then decreases, and the band gap center frequency changes little. In particular, the change of $h_{1}/h$ will not only change the width of the band gap but also will change the stiffness of the supporting beam of the structure. The results show that when 0.1 < $h_{1}/h$ < 0.2 and 0.35 < $a_{1}/a$ < 0.40, the center frequency is in the UHF range and the bandwidth reaches more than 250 MHz.

## 4. LWR Design and Analysis Results

### 4.1. LWR Design

**Figure 6:**
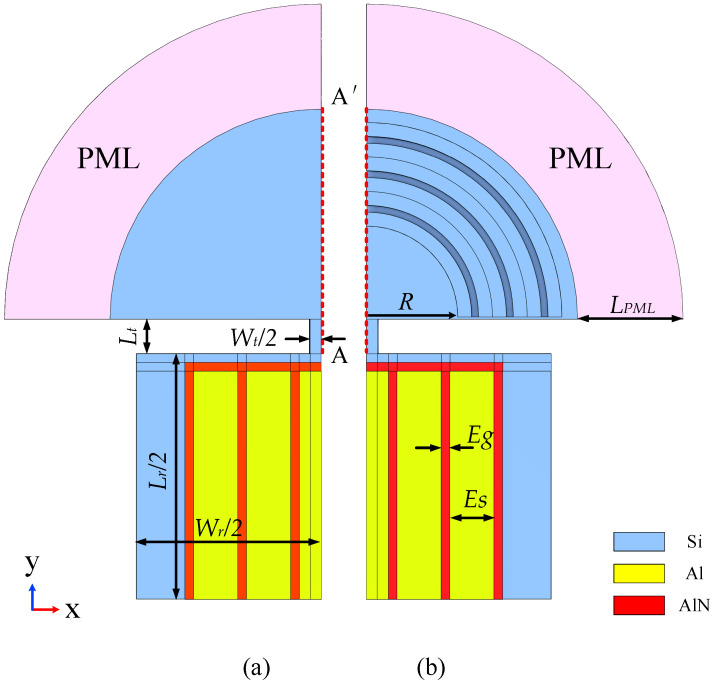
LWR model. (**a**) conventional simplified 1/4 model; (**b**) LWR 1/4 simplified model after adding three cycles of H-RPC.

The resonators designed in this paper are all LWRs, and the simplified model is shown in Figure 6. Figure 6a shows the simplified 1/4 model of a conventional LWR, and Figure 6b shows the simplified LWR 1/4 model after adding three cycles of H-RPC.

The width extended (WE) vibration mode expression of the LWR is [40]:

(5) $$\begin{array}{c} f_{r}=\frac{nv}{2W_{r}} \end{array}$$

In Equation (5), v represents the sound velocity in the resonator, $W_{r}$ is the width of the rectangular resonator, and n represents that the resonator has an *n*-order harmonic mode. In this paper, we study the rectangular resonator *n* with order seven. The resonant frequency $f_{r}=349.08\;\mathrm{MHz}$. The specific size parameters of the resonators are shown in Table 3. Through the input power of the yellow electrode region (Al) of the resonator in Figure 6, the red piezoelectric layer region (AlN) performs positive and inverse piezoelectric effects to drive the cadet blue substrate (Si) to vibrate, and then the middle electrode of the resonator is used for output power. The material used in the substrate Si is consistent with the material used in H-RPC. In addition, the material parameters of Al and AlN are shown in Table 4.

In addition, in order to further verify the effect of applying H-RPC resonators to reduce anchor loss, PCs periodic structures with different *R* (0 μm, 5 μm, 10 μm, and 15 μm) spacings are applied on the anchor of the resonator as shown in Figure 6. In this study, the simulation model of the resonator absorbs the dissipated elastic wave according to PML ($L_{PML}=3\times \lambda$). This study ignores other factors and only considers the main anchor loss factors [38]. The anchor quality factor ($Q_{anc}$) of the resonator can be obtained from [41]:

(6) $$\begin{array}{c} Q_{anc}=\frac{Re\left(\omega \right)}{2lm\left(\omega \right)} \end{array}$$

In Equation (6), Re(ω) represents the real part of the resonant angular frequency of the resonator, and lm(ω) represents the imaginary part of the resonant angular frequency of the resonator.

**Figure 7:**
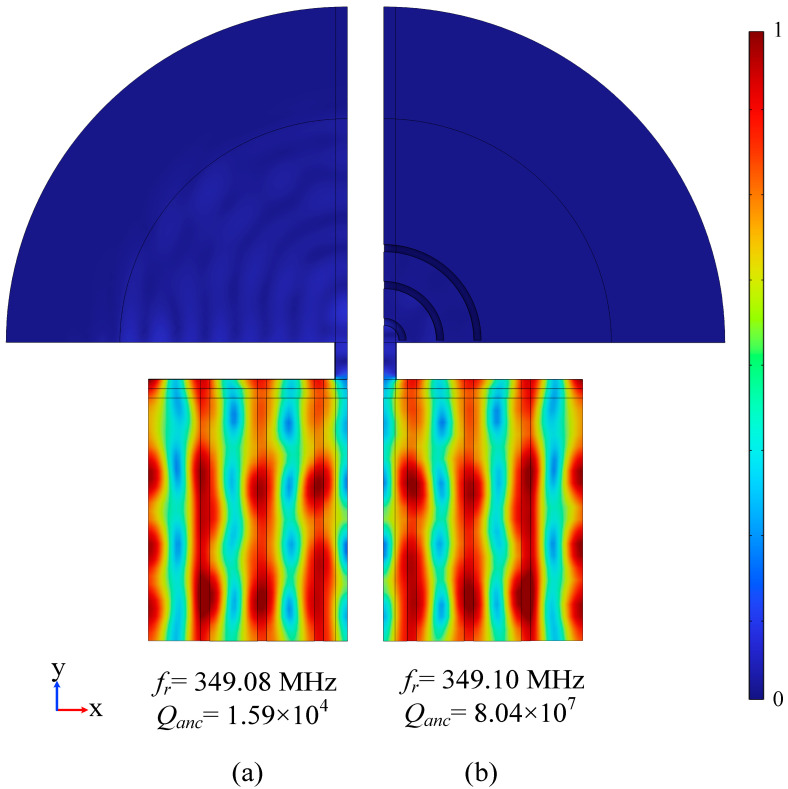
Finite element simulation of resonance mode. (**a**) Conventional LWR mode 1/4; (**b**) LWR mode 1/4 with three cycles of H-RPC and R = 0. $f_{r}$ is the resonant frequency and $Q_{anc}$ is the quality factor of the anchor.

Figure 7 shows the modal diagram of the finite element simulation results. Figure 7a is the resonant mode of the conventional LWR. The resonant frequency is $f_{r}=349.08\;\mathrm{MHz}$ and $Q_{anc}=1.59\times 10^{4}$. Figure 7b is the LWR mode diagram when *R* = 0μm after adding H-RPC. The resonant frequency is $f_{r}=349.10\;\mathrm{MHz}$ and $Q_{anc}=8.04\times 10^{7}$. In addition, when *R* = 5 μm, the resonant frequency $f_{r}$ is 349.10 MHz and $Q_{anc}=3.82\times 10^{6}$; when *R* = 10 μm, the resonant frequency $f_{r}$ is 349.11 MHz and $Q_{anc}=1.08\times 10^{7}$; when *R* = 15 μm, the resonant frequency $f_{r}$ is 349.04 MHz and $Q_{anc}=6.07\times 10^{6}$. The results show that the $Q_{anc}$ is significantly improved after adding H-RPC. When *R* = 0 μm, $Q_{anc}$ is increased to $8.04\times 10^{7}$, which is 505560.4% higher than that of the conventional LWR.

### 4.2. Analysis Results

**Figure 8:**
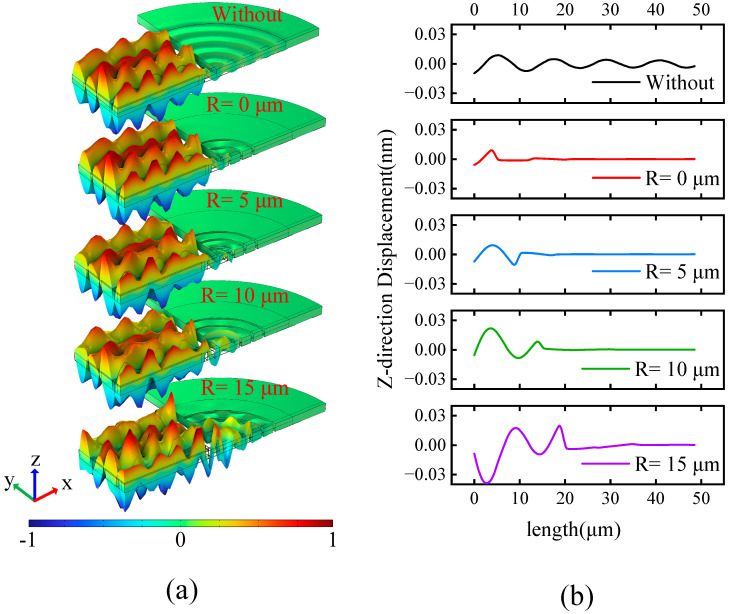
(**a**) The vibration mode of the resonator in the resonant mode; (**b**) The Z-direction of the A-A‘ is the displacement diagram.

Figure 8 shows the result diagram of the resonator at the resonant frequency. Figure 8a represents the modal diagram of the resonator at the resonant frequency, and Figure 8b represents the displacement diagram of the resonator‘s section A-A‘ at the resonant frequency. From the Z-direction vibration mode diagram of the conventional LWR in Figure 8a, it can be seen that the elastic wave after the support tether radiates outward with ripples, and the elastic wave can be added after the H-RPC structure is added. It can be seen from Figure 8b that when *R* = 0 μm, the elastic wave has the greatest suppression effect before reaching the first wave peak; when *R* = 5 μm, the elastic wave has an effective suppression effect before reaching the first wave trough; when *R* = 10 μm, the elastic wave has an effective suppression effect after experiencing a periodic waveform, and it can be clearly observed that the waveform displacement of the first cycle is higher than without H-RPC the periodic waveform. It is worth noting that when *R* = 15 μm, the waveform displacement of the elastic wave is oppositely excited, and the reflected acoustic energy has a certain influence on the main resonant mode of the resonator. The results show that when H-RPC is closer to the support tether it is more conducive to suppressing the anchor loss of the resonator.

In addition, under the load of 50-Ω, the admittance Y11 curve and the insertion loss curve of the resonator are calculated by finite element frequency domain simulation. The Q and electromechanical coupling coefficient of the resonator under load are further calculated. The Q and electromechanical coupling coefficient ($k_{eff}^{2}$) are obtained according to the following formula [42,43]:

(7) $$\begin{array}{c} Q=\frac{f_{s}}{\Delta f_{-3dB}},k_{eff}^{2}=\frac{f_{p}^{2}-f_{s}^{2}}{f_{p}^{2}} \end{array}$$

Among them, $\Delta f_{-3dB}$ is the 3 dB bandwidth of the series resonator, $f_{s}$ is the series resonance frequency of the resonator, and $f_{p}$ is the parallel resonance frequency of the resonance.

**Figure 9:**
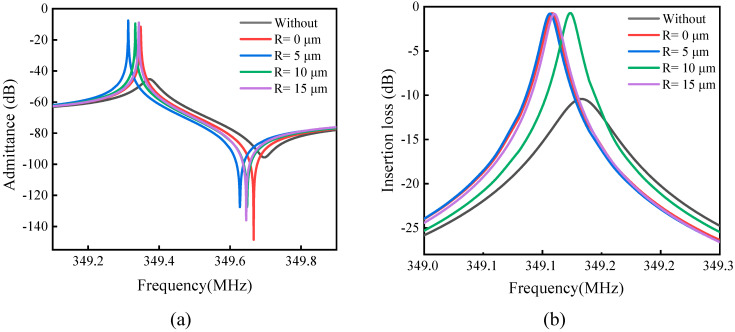
(**a**) admittance curve Y11; (**b**) insertion loss curve.

The results are shown in Figure 9, where Figure 9a shows the admittance curve Y11 and Figure 9b shows the insertion loss curve. Figure 9 contains the data results of conventional LWR results and different R values after adding the H-RPC. When using a conventional LWR, Q=2773, $k_{eff}^{2}=0.18\%$, and insertion loss is 10.43 dB. After adding the H-RPC when *R* = 0 μm, Q=8734, $k_{eff}^{2}=0.18\%$, and insertion loss is 0.74 dB; when *R* = 5 μm, Q=7939, $k_{eff}^{2}=0.18\%$, and insertion loss is 0.80 dB; when *R* = 10 μm, Q=9441, $k_{eff}^{2}=0.18\%$, and insertion loss is 0.72 dB; when *R* = 15 μm, Q=9704, $k_{eff}^{2}=0.17\%$, and insertion loss is 0.81 dB. When R = 5 μm, the Q exhibits maximum improvement, increasing from 2773 to 9704, which is 249.9% higher than that of the conventional LWR. The electromechanical coupling coefficient of the LWR is between 0.17% and 0.18%, and the addition of the H-RPC has little effect on the electromechanical coupling coefficient. When *R* = 10 μm, the insertion loss decreases from 10.43 dB to 0.72 dB, which is 93.10% lower than that of the conventional LWR. The results show that the LWR with the H-RPC can increase the Q by up to 249.9%, and the insertion loss can be reduced by up to 93.10%. At the same time, the addition of the H-RPC has little effect on the electromechanical coupling coefficient of the LWR.

In order to measure the performance of the LWR more intuitively, the Figure of Merit (FOM) between resonators can be compared. A standard definition of FOM for resonators is [44]:

(8) $$\begin{array}{c} FOM=\frac{k_{eff}^{2}\;\cdot \;Q}{1-k_{eff}^{2}} \end{array}$$

The FOM results are shown in Table 5. It can be seen from Table 5 that the FOM can be increased from the original 5.0 to 15.7, 14.3, 17.0, and 16.5 by adding the H-RPC. When R = (0 μm–10 μm), the Q is significantly improved, and the coupling coefficient does not change. When R continues to increase to R = 15 μm, although the Q shows the largest increase, due to the decrease of the coupling coefficient, the FOM is reduced by 0.5 compared to 17.0 at R = 10 μm. Therefore, when R = 10 μm, the performance of the resonator is optimal, and the FOM can reach 2.4 times the original.

## 5. Conclusions

This paper studies an H-RPC structure based on cylindrical coordinates, which has an ultra-wideband gap. Using the finite element method, through the analysis of the complex band curve and frequency response, the width of the ultra-wideband gap is as high as 238 MHz, and the relative bandwidth is as high as 80.2%. The attenuation of the H-RPC structure in a finite period is studied. When the H-RPC structure has four periods and the frequency is 400 MHz, the attenuation can reach 55 dB. The influence of geometric parameters on the ultra-wideband gap is further studied. The results show that the change of $h_{1}/h$ has a great influence on the center frequency and bandwidth of the band gap, while the change of $a_{1}/a$ only has a great influence on the bandwidth. When 0.1 < $h_{1}/h$ < 0.2 and 0.35 < $a_{1}/a$ < 0.40, the center frequency is in the UHF range and the bandwidth reaches more than 250 MHz.

In addition, the H-RPC structure can significantly improve the anchor loss of an LWR in the UHF range. When the H-RPC structure spacing *R* = 0 μm, the $Q_{anc}$ is increased to 8.04 × 10^7^, which is 505560.4% higher than that of the conventional LWR. In addition, the quality factor, electromechanical coupling coefficient, and insertion loss under load are studied. When the H-RPC is added, the Q and insertion loss of the LWR are significantly improved. The Q is increased by 249.9% at most and the insertion loss is reduced by 93.10% at most, while after adding the H-RPC the LWR does not have a great influence on the electromechanical coupling coefficient. When R = 10 μm, the performance of the resonator is optimal, and the FOM can reach 2.4 times the original. This study provides a new idea for improving the performance of LWR, and further future experimental work will be required to fully validate the modelled results.

## Figures and Tables

**Table 1:** Parameters of Si Materials.

Elastic Modulus	Shear Modulus	Poisson’s Ratio
$E_{x}=169\;GPa$	$G_{xy}=50.9\;\mathrm{GPa}$	$\sigma_{xy}=0.064$
$E_{y}=169\;\mathrm{GPa}$	$G_{yz}=79.6\;\mathrm{GPa}$	$\sigma_{yz}=0.36$
$E_{z}=130\;\mathrm{GPa}$	$G_{zx}=79.6\;\mathrm{GPa}$	$\sigma_{zx}=0.28$

**Table 2:** Band gap comparison of similar lattice constants.

Reference	Lattice Constant (μm)	Band Gap Range (MHz)	Center Frequency (MHz)	Bandwith (MHz)	BG
[3]	4	472–601	536	129	24.0%
[13]	10	180–340	260	160	61.5%
[16]	8	562–624	593	62	10.5%
This work	8	209.4–489.7	349	280	80.2%

**Table 3:** The size parameters of the resonator.

Symbol	Dimension	Values
λ	Wave length	24.3 μm
W_r_	Resonator width	85 μm
L_r_	Resonator length	113.4 μm
W_t_	Tethers Width	5 μm
L_t_	Tethers Length	8 μm
E_g_	Electrode gap	2 μm
E_s_	Electrode spacing	10.2 μm
T_Al_	Thickness of Al	0.5 μm
T_AlN_	Thickness of AlN	0.5 μm
T_Si_	Thickness of substrate Si	5 μm

**Table 4:** Parameters of Al and AlN Materials.

Materials	Parameters	Values
Aluminum Nitride (AIN)	Density (ρ)	3300 kg/m^3^
	Relative permittivity (ε)	9
	Poisson’s ratio (ν)	0.24
	Young’s Modulus (E)	320 Gpa
Aluminum (Al)	Density (ρ)	2700 kg/m^3^
	Young’s Modulus (E)	70 Gpa
	Poisson’s ratio (ν)	0.35
	Electrical conductivity (σ)	35.5 × 10^6^ S/m
	Coefficient of thermal expansion (α)	23.1 × 10^−6^/K
	Heat capacity (Cp)	904 J/Kg K
	Thermal conductivity (κ)	237 W/mK

**Table 5:** FOM comparison after adding H-RPC.

Parameters		$k_{eff}^{2}$	Q	FOM
without		0.18%	2773	5.0
H-RPC	R=0 μm	0.18%	8734	15.7
	R=5 μm	0.18%	7939	14.3
	R=10 μm	0.18%	9441	17.0
	R=15 μm	0.17%	9704	16.5

## Data Availability

All data needed to evaluate the conclusions in the paper are present in the paper.
